# Identification of the DNA-Binding Domains of Human Replication Protein A That Recognize G-Quadruplex DNA

**DOI:** 10.4061/2011/896947

**Published:** 2011-05-21

**Authors:** Aishwarya Prakash, Amarnath Natarajan, Luis A. Marky, Michel M. Ouellette, Gloria E. O. Borgstahl

**Affiliations:** ^1^The Eppley Institute for Research in Cancer and Allied Diseases, University of Nebraska Medical Center, 987696 Nebraska Medical Center, Omaha, NE 68198-7696, USA; ^2^Department of Pharmaceutical Sciences, College of Pharmacy, University of Nebraska Medical Center, Omaha, NE 68198-6025, USA

## Abstract

Replication protein A (RPA), a key player in DNA metabolism, has 6 single-stranded DNA-(ssDNA-) binding domains (DBDs) A-F. SELEX experiments with the DBDs-C, -D, and -E retrieve a 20-nt G-quadruplex forming sequence. Binding studies show that RPA-DE binds preferentially to the G-quadruplex DNA, a unique preference not observed with other RPA constructs. Circular dichroism experiments show that RPA-CDE-core can unfold the G-quadruplex while RPA-DE stabilizes it. Binding studies show that RPA-C binds pyrimidine- and purine-rich sequences similarly. This difference between RPA-C and RPA-DE binding was also indicated by the inability of RPA-CDE-core to unfold an oligonucleotide containing a TC-region 5′ to the G-quadruplex. Molecular modeling studies of RPA-DE and telomere-binding proteins Pot1 and Stn1 reveal structural similarities between the proteins and illuminate potential DNA-binding sites for RPA-DE and Stn1. These data indicate that DBDs of RPA have different ssDNA recognition properties.

## 1. Introduction

**Figure 1:**
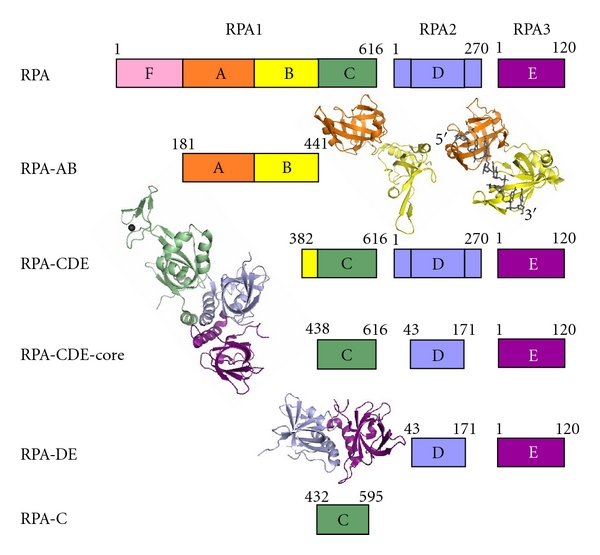
RPA domain structure, constructs used in these studies and their corresponding crystal structures. RPA-AB contains RPA1_181-441_. RPA-CDE includes RPA1_382-616_ and intact RPA2 and RPA3. RPA-CDE-core consists of RPA1_438-616_, RPA2_43-171_, and intact RPA3. RPA-DE includes RPA2_43-171_ and intact RPA3. RPA-C is composed of RPA1_432-595_ with 7 point mutations (V435T, W442Q, V465T, V469T, F523S, F567S, and I571T). Crystal structure representations of RPA-AB apo, RPA-AB + dC_8_, RPA-CDE-core, and RPA-DE are shown next to their domain maps [6–9].

Heterotrimeric replication protein A (RPA) is the primary eukaryotic single-stranded DNA- (ssDNA-) binding protein [1–3]. The three subunits are named RPA1 (70 kDa), RPA2 (32 kDa), and RPA3 (14 kDa) (Figure 1). RPA is a central player in all aspects of DNA metabolism, and it is thought to have little sequence specificity. RPA is a modular protein composed of several domains connected by flexible linkers, and it undergoes a conformational change upon ssDNA binding [4]. RPA is thought to assume a variety of structures depending on the nature of the DNA substrate [5]. This paper seeks to understand if RPA and its individual DNA-binding domains (DBDs) can selectively recognize any unique DNA sequences. 

RPA binds ssDNA with high affinity (K_a_ ~ 10^9^–10^11^ M^−1^) and low cooperativity and binds polypyrimidine sequences with higher affinity than polypurine sequences [1, 13–15]. RPA contains six oligonucleotide binding (OB) folds (named A-F), five of which have previously been shown to possess DNA-binding activity (A-E) [1, 16] (Figure 1). These DBDs have been proposed to bind DNA in a sequential fashion in which DBD-A and -B (RPA-AB) contact 8-nt of DNA [K_a_ ~ 10^6^–10^8^ M^−1^]  depending on the size and nature of the sequence used [5, 17]. Addition of DBD-C is needed to bind a 12–23 nt ssDNA fragment, and DBD-D completes the footprint, allowing binding to 25–30 nt [5, 16]. DBD-A and -B are in the middle of RPA1 and are required for high affinity binding of RPA to ssDNA (Figure 1) [14, 18]. DBD-C located near the C-terminus of RPA1 contains a zinc-finger motif within the OB-fold, binds ssDNA with lower affinity, and is required for heterotrimeric complex formation [19, 20]. DBD-C binds specifically to a pyrimidine-(6-4)-pyrimidone photoproduct and requires the presence of zinc [19]. DBD-D is in the center of RPA2 (Figure 1), has a similar low affinity for ssDNA, and is also involved in the formation of the heterotrimer [21]. DBD-E in RPA3 has an OB-fold that is primarily known for its subunit interactions. Recent photo-crosslinking experiments suggested that DBD-E can also bind, albeit transiently and with low affinity, the 3′-end of ssDNA molecules bound to RPA [22, 23]. DBDs-C, -D, and -E (RPA-CDE) form a trimer core that can recognize and bind to a primer-template junction [24]. Most of the analyses of RPA's interaction with ssDNA are based on studies of the interaction of the protein with poly-purine and poly-pyrimidine sequences. Recently, however, more light has been shed on the interaction of RPA with mixed ssDNA sequences [25] as well as noncanonical ssDNA sequences capable of forming secondary structures such as triplexes and G-quadruplexes [26–29]. In contrast to *E. coli* and T4 ssDNA-binding proteins, RPA can melt DNA triplexes and depletion of RPA in HeLa cells caused triplex DNA content to increase [30]. Native gel electrophoresis, cross-linking, and fluorescence resonance energy transfer experiments indicate that RPA can bind and unfold a 21-mer telomeric G-quadruplex sequence [26]. Most recently, studies employing circular dichroism (CD) indicate that RPA can bind and unfold intramolecular G-quadruplex structures [29]. Taken together, these studies indicate a role for RPA in binding noncanonical ssDNA structures. 

G-quadruplex DNA interactions with RPA are of great interest because of the capability of a vast number of sequences in the human genome to form G-quadruplexes [31]. G-quadruplexes result from the stacking of G-quartets which form when four planar guanine residues interact via Hoogsteen hydrogen bonds [32, 33]. Sequences with G-quadruplex forming potential are found throughout the genome and at the ends of telomeres and must be unfolded for accurate DNA replication [32]. RPA helps prevent the accumulation of telomeric DNA in cells employing alternative lengthening of telomeres [34], supports telomerase activity in yeast [35, 36], restores human telomerase activity *in vitro *[37], and causes telomere shortening in human cancer cells [38]. Also, G-rich sequences capable of forming secondary structures were identified upstream of transcriptional promoters in *S. cerevisiae* and were shown to regulate transcription in cells exposed to G-quartet stabilizers [39] In human cells, sequences with the propensity to form quadruplexes have been implicated in the transcriptional regulation of promoters of the c-myc, HIF-1*α*, bcl-2, and c-kit oncogenes [31], making these G-rich sequences intriguing and important to study. 

Several proteins/ligands bind to, and promote or unfold, G-quadruplex structures in DNA [40]. Among these, is the Pot1 (protector of telomeres 1) protein that contains two N-terminal OB-folds that bind human telomeric G-rich DNA [41, 42]. The crystal structure of the N-terminal OB-folds of Pot1 bound to a telomeric ssDNA sequence 5′-TTAGGGTTAG-3′ indicates that these OB-folds adopt an elongated conformation where the OB-folds pack in tandem creating a single continuous channel with a kink at the interface between the OB-folds [43]. This is different than the arrangement seen in the crystal structure of RPA-AB bound to ssDNA (dC_8_) where the loops of the OB-folds form a channel that extends from DBD-A to DBD-B yielding tight DNA binding (Figure 1, RPA-AB structures) [6]. In yeast, an RPA2/RPA3-like complex of Stn1/Ten1 interacts specifically with telomeric DNA [28, 44]. Stn1/Ten1 use OB-fold structures, much like the OB-folds of RPA, to contact DNA. In fact, a superposition of Stn1 and RPA2 displays a great deal of structural homology between the two OB-folds [45]. Superpositions of RPA DBDs-A, -B, -C, and -D with Pot1 DBDs OB-1 and OB-2 indicate that RPA2 has the most structural similarity to Pot1 (Prakash, unpublished). This raises the possibility that some of RPA's OB-folds, such as DBD-D of RPA2, may display some sequence specificity and possibly a preference for G-rich DNA. 

Systematic Evolution of Ligands by EXponential enrichment (SELEX) methodology has been used successfully to define the sequence specificity of DNA-binding proteins. The first SELEX study on a ssDNA-binding protein was performed with the bacteriophage Ff gene 5 protein [46]. Gene 5 protein, is an OB-fold containing protein that binds and sequesters nascent viral ssDNA prior to packaging the DNA into virions. Therefore, it was originally thought to bind nonspecifically to ssDNA but SELEX revealed a binding preference to a G-rich, G-quadruplex forming DNA sequence [46, 47]. In the study reported here, SELEX was used to detect specific ssDNA by RPA-CDE. The secondary structure of ssDNA and the ability of RPA's DBDs to unfold ssDNA were monitored with CD experiments. These data, in combination with fluorescence polarization (FP) DNA-binding studies, help explain the complexity of how the various DBDs of RPA orchestrate and contribute to the binding of RPA to numerous DNA sequences.

## 2. Materials and Methods

### 2.1. RPA Constructs and Purification Scheme

 Plasmids for full-length human RPA (Figure 1) and the RPA-CDE construct were obtained from Dr. Marc Wold, University of Iowa. Plasmids of RPA-CDE-core and RPA-DE were obtained from Dr. Walter Chazin, Vanderbilt University. RPA-AB was cloned into a pET28a vector with an N-terminal 6-His-tag for over expression in *E. coli*. An RPA-C construct (RPA1 residues 432-595) with an N-terminal His-tag was created in a pET28a vector with the following point mutations: V435T, W442Q, V465T, V469T, F523S, F567S, and I571T. 

Overexpression in bacteria followed standard procedures. The purification scheme of RPA and RPA-CDE followed previous protocols where the proteins were purified by fractionation over Affi-gel Blue, Hydroxyapatite, and Mono-Q columns [48, 49]. RPA-AB, RPA-CDE-core, and RPA-DE all contained thrombin-cleavable, N-terminal His-tags and were purified using Nickel column chromatography and tag cleavage, followed by Hydroxyapatite chromatography or Mono-Q anion exchange chromatography. For RPA-C, the protein was purified by solubilizing inclusion bodies with 4 M guanidinium hydrochloride in Buffer-B (25 mM Tris, pH 8, 2 M urea, 250 mM NaCl, 10 *μ*M ZnCl_2_, 20 mM imidazole, and 2 mM *β*-mercaptoethanol). Using nickel column chromatography, the protein was refolded on the column and eluted with an imidazole gradient in Buffer-B. Thrombin (Sigma) was used to cleave the His-tag, and the protein was passed over nickel resin a second time to remove any remaining contaminants. The gels of purified proteins are provided (Supplementary Figure S1 available online at doi:10.4061/2011/896947). Proteins were concentrated by ultrafiltration and concentrations were determined using the absorbance at 280 nm. The extinction coefficients (*ε* = M^−1^cm^−1^) and M_W_ are listed as follows: RPA, *ε* = 88 × 10^3^, M_W_ = 110 kDa; RPA70-AB (no His-tag), *ε* = 32.08 × 10^3^, M_W_ = 27.07 kDa; RPA-CDE, *ε* = 55.25 × 10^3^, M_W_ = 69.365 kDa; RPA-CDE-core, *ε* = 37.35 × 10^3^, M_W_ = 49.069 kDa; RPA70-C, *ε* = 16.65 × 10^3^, M_W_ = 18.59 kDa; RPA14/32core, *ε* = 24.9 × 10^3^, M_W_ = 27.851 kDa. These coefficients were calculated based on the amino acid sequence using DNASTAR software.

### 2.2. SELEX Procedure

 A synthesized random-core library of 75-mer ssDNA was used for SELEX. This sequence contained a 35-nt random core, flanked by 20-nt PCR priming sites, and was synthesized with the following sequence: 5′-CAGTAGCACACGACATCAAG-N_35_-GCATGTCTCGTGTCAGTTG-3′. The nucleobases A, G, C, and T were randomly incorporated during chemical synthesis of the central 35-nt. The 35-nt random core used here for SELEX was advantageous since the random core is slightly larger than the known footprint of a single RPA trimer (~28–30 nt). The sequence of the forward and reverse PCR primers was as follows: 5′-CAGTAGCACACGACATC-3′ and 5′-CAACTGACACGAGACAT-3′. For the initial selection, 1 nmol (26 trillion sequences) of the oligo pool was incubated for 30 minutes with 50 ng of RPA-CDE and 2 *μ*g of competitor *E. coli* DNA, in 20 *μ*L of binding buffer containing 4% glycerol, 1 mM MgCl_2_, 0.5 mM EDTA, 0.5 mM DTT, 50 mM NaCl, and 10 mM Tris-HCl, pH 7.5 (Promega gel shift binding buffer). Protein: DNA complexes were pulled down using magnetic beads (M450 Dynabeads) coated with anti-RPA2 antibody (Oncogene) at room temperature. The beads were then washed with binding buffer and resuspended in a PCR mix containing 1X Taq buffer, 200 *μ*M dNTP, 1.5 mM MgCl_2_, 1 *μ*M of each primer, and platinum Taq-polymerase (Invitrogen). The reaction was first heated to 95°C to remove the beads, and then the DNA was subjected to 30 cycles of PCR (95°C for 1 min, 56°C for 1 min, and 72°C for 2 min). Aliquots of 20 *μ*L were removed every 5 cycles until 30 cycles were completed and separated on a 3% agarose gel. The band that corresponded to 75-bp was cut out and gel purified. The eluted DNA was reamplified for 16 cycles in a PCR mix containing only the forward primer, and the ssDNA obtained was then ethanol precipitated and resuspended in binding buffer for use in subsequent rounds of SELEX. A total of 6 rounds of SELEX was performed. The PCR product from the last round of SELEX was cloned into TOPO 2.1 vector (Invitrogen) and transformed into DH5*α* cells. Transformants were selected by ampicillin resistance supplemented with X-Gal and IPTG for blue/white screening. A total of 30 sequences were obtained and analyzed for any consensus.

### 2.3. Circular Dichroism (CD) Experiments of Oligonucleotides and Protein:ssDNA Complex Formation

 An Aviv CD spectrometer Model 202SF equipped with a Peltier temperature control system (Lakewood, NJ) was used to characterize the conformation of each protein, oligomer and complex. Sample solution was placed in a strain-free quartz cell, and the spectrum was recorded every 1 nm. All spectra were recorded in Buffer A which contained 25 mM Tris pH 7.5, 2 mM MgCl_2_, 6% glycerol, 1 mM DTT, and 100 mM NaCl. The buffer only curve was subtracted and then normalized for concentration and dilution effects. Data recorded were averages of 3 scans. For DNA CD spectra, 2 *μ*M ssDNA with the following sequence 5′-dTAGGGGAAGGGTTGGAGTGGGTT-3′ called Gq23 was placed in a 1 cm CD cell. Spectra were recorded at varying temperatures: 10, 20, 40, 60, and 80°C. For protein CD spectra, ~10 *μ*M of each protein was placed in a 0.1 cm cell, and spectra were recorded from 190 to 240 nm. For titration of ssDNA at varying protein:ssDNA molar ratios (0–8), spectra were recorded in a 1 cm cuvette. As a control to ensure properly folded proteins, spectra were collected on all purified proteins at varying temperatures (4°C, 25°C and 37°C; Supplementary Figure S2). Deconvolution of the spectra was performed using Dichroweb algorithm CDSSTR [50] (Supplementary Figure S2(a)–(c) and attached discussion). All proteins appeared to have been folded properly as they displayed secondary structures in good agreement with the available published structures [6–8].

### 2.4. Preparation of Oligonucleotides

 Synthetic oligonucleotides were prepared for CD and FP experiment by thermal equilibration, and the folds of the oligonucleotides were monitored by CD. For Gq23 the rate of equilibration had no affect so Gq23 was quickly heated to 85°C and cooled rapidly to 2°C. In both cases, spectra were recorded upon raising the temperature to 25°C, since all titration experiments with proteins were carried out at room temperature.

### 2.5. Fluorescence Polarization (FP) Binding Assays

All ssDNA-protein binding interactions were carried out using FP as described previously [51]. This assay measures the change in FP of a fluorescently-labeled ssDNA in the presence of a binding protein. The fluorescent species is excited using plane polarized light. The molecule rotates and tumbles out of this plane during the excited state and results in the emission of light in a different plane. FP measured (see (1)) is proportional to the tumbling rate which correlates with the average molecular size of the fluorescent species

(1) $$\text{FP}=\frac{{\text{I}}_{||}-{\text{I}}_{⊥}}{{\text{I}}_{||}+{\text{I}}_{⊥}},$$

where I_||_ = Intensity with polarizers parallel, I_⊥_ = Intensity with polarizers perpendicular, and the instrument correction factor is automatically included in the output from the instrument. A binding isotherm is generated by adding increasing amounts of protein to a constant amount of ssDNA. In a typical competition assay, the unlabeled oligo is titrated into a mixture that has a constant amount of labeled ssDNA and protein. In both cases, the concentration of the variant is plotted against a change in FP. The ssDNA sequences used for FP (Integrated DNA Technologies) and were labeled with 5′ Fluorescein (6-FAM) followed by an 18-carbon spacer (sp18) placed on the 5′ end of the sequence. The spacer was needed since the G-rich sequences folded into complex quadruplex structures that quenched the FAM signal. With the space in place, FAM placed at the 5′ end of the spacer could be easily detected. The sequences used were as follows: 

Gq23 5′-6-FAM-sp18-TAGGGGAAGGGTTGGAGTGGGTT-3′

Anti 5′-6-FAM-sp18-ATCCCCTTCCCAACCTCACCCAA-3′

PolyA 5′-6-FAM-sp18-AAAAAAAAAAAAAAAAAAAAAAA-3′

PolyG 5′-6-FAM-sp18-AAAGGGGGGGGGGGGGGGGGGGG-3′

Reactions (10 *μ*L) were assembled at room temperature in buffer A in a black 384-well Corning round-bottom, low volume plate for all measurements. The NaCl concentration in buffer A was varied from 10 to 1500 mM depending on the protein construct being studied. FP measurements were recorded at an excitation wavelength of 485 nm and emission of 535 nm using an M5 SpectraMax multimode microplate reader (Molecular Devices). Plots of FP versus protein concentration were generated using SigmaPlot 11, and dissociation-binding (*K*_*d*_) constants were obtained by fitting the data using standard 4-parameter logistic curve defined below

(2) $$Y=\min\;+\frac{\max\;-\min\;}{1+{\left(x/K_{d}\right)}^{-\text{slope}}}.$$

Competition assays with RPA, RPA-CDE-core, and Anti ssDNA were performed by titrating the protein bound to labeled ssDNA with unlabeled competitor ssDNA. The results indicate that binding of RPA to the labeled ssDNA sequences is not due to the label but specific to the ssDNA sequence (Supplementary Figure S3).

## 3. Results

### 3.1. Measurement of RPA-CDE's ssDNA Sequence Specificity

**Figure 2:**
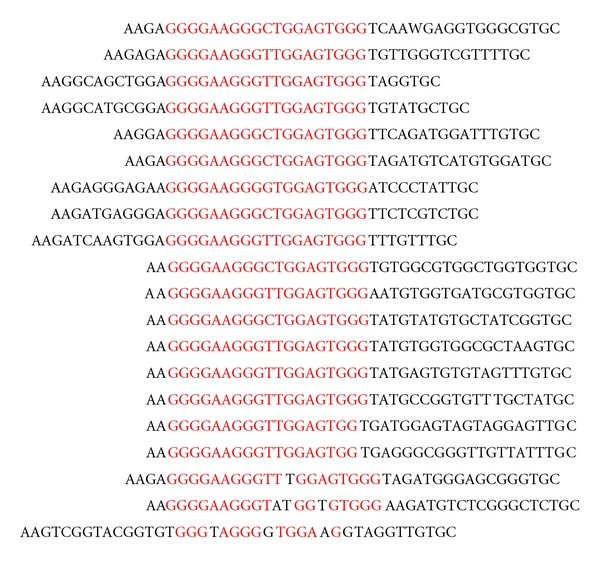
The consensus sequence obtained for RPA-CDE after SELEX, GGGGAAGGGYTGGAGTGGGT (Y = T/C), was present in 63% of all sequences analyzed. Red letters indicate alignment from the trinucleotide analysis.

 SELEX was used to examine the DNA-binding specificity of RPA-CDE. The high-affinity RPA-binding sites from a pool of randomized ssDNA molecules were selected by immunoprecipitation of RPA/DNA complexes. After six successive rounds of selection, the selected sites were cloned and sequenced. To help search for consensus motifs, the selected DNA were analyzed for the occurrence of each of the 64 possible trinucleotides. Trinucleotides that were found to be overrepresented were then used to search for a larger consensus occurring in the majority of the selected oligonucleotides (Supplementary Figure S4). Preliminary analysis of the original unselected random pool revealed a slight bias for G-rich sequences as has been previously reported for randomly synthesized DNA [52]. SELEX using RPA did not reveal any sequence specificity (Prakash unpublished, and [29]). Selection with RPA-CDE produced striking results. Here, 63% of the 32 cloned sequences contained the G-rich motif GGGGAAGGGYTGGAGTGGGT (Y = C/T) (Figure 2). These results were very different from the known preference of full length RPA for pyrimidines and were explored further.

### 3.2. G-Rich SELEX Oligonucleotide Forms a G-Quadruplex Structure

**Figure 3:**
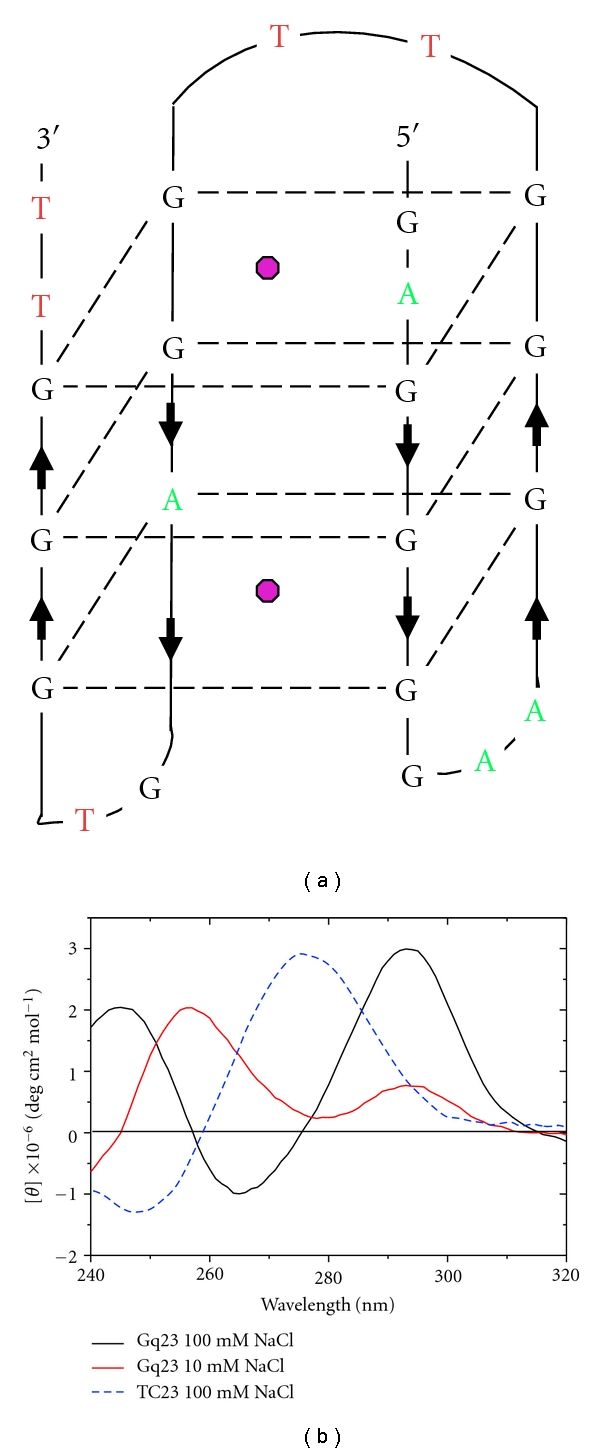
CD characterization of the Gq23 and TC23 oligonucleotides. (a) Antiparallel model of Gq23 (G black, T red, and A green). Note, the third G-quartet stack (from the top) contains an A instead of the canonical G. Pink circles indicate sodium ions that aid in G-quartet formation and stacking. This hypothetical model shows just one of several possible base pairing arrangements for a Gq23 intramolecular quadruplex. (b) Spectra of Gq23 recorded in a 1 cm cuvette at 25°C in 100 mM NaCl (black), 10 mM NaCl (red), and the TC23 sequence in 100 mM NaCl (dashed blue).

 The G-rich consensus motif selected by RPA-CDE, Gq23, was modeled to fold into a G-quadruplex with three potential G-quartets, including one with a nonguanine base (dATP substituted for dGTP; Figure 3(a)). CD spectroscopy was used to study the secondary structure of Gq23. Spectra taken in 100 mM NaCl buffer on a 23-nt oligonucleotide with the sequence 5′-dTAGGGGAAGGGTTGGAGTGGGTT-3′, termed Gq23, had a maximum absorption peak at 292 nm. This is indicative of an antiparallel conformational arrangement of the bases involved in the formation of the G-quartet stacks (Figure 3(b) black line, Figure 6(a)). The independence of melting temperature, T_M_, with strand concentration demonstrated that the G-quadruplex was intramolecular at both 10 and 100 mM NaCl (data not shown). Thus, in a buffer containing 100 mM NaCl, Gq23 forms an antiparallel, intramolecular G-quadruplex. 

As some FP DNA binding studies were done at different salt concentrations, the effect of salt on ssDNA conformation was measured. At 10 mM NaCl, the conformation of Gq23 changes and favors the parallel form (peak at ~254 nm) over the antiparallel conformation (Figure 3(b) red line, Figure 6(c)). As a control, a 23-nt oligomer, TC23 with the sequence 5′-dGTCTTCCTTAATTGTCTTCCTTA-3′ was analyzed. TC23 contained 2 repeats of the 8-mer consensus selected by RPA-AB. As expected, TC23 forms a random coil (with characteristic crossover at 260 nm and a peak at 280 nm) with no secondary structure (Supplemental Figure S5(a), Figure 3(b) blue dashed line).

### 3.3. Deconvolution of the Binding Affinity of RPA Domains to Various ssDNA Sequences

**Figure 4:**
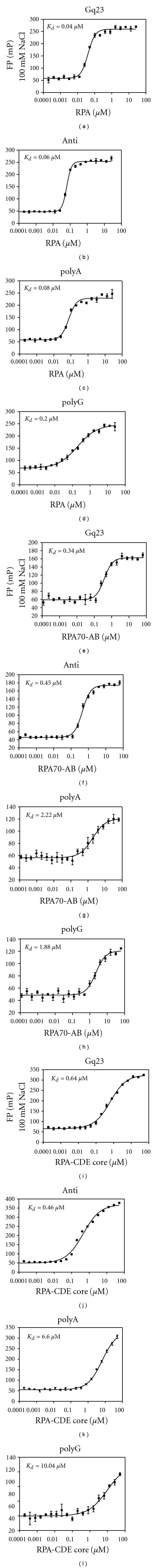
FP ssDNA-binding assays for RPA, RPA-AB, and RPA-CDE-core at 100 mM NaCl. Representative binding isotherms are shown. (a)–(d) RPA, (e)–(h) RPA-AB, and (i)–(l) RPA-CDE-core. The ssDNA sequences (Gq23, Comp, polyA, or polyG) are displayed at the top of each column, and the protein construct is listed at the beginning of a row. For each FP reaction, 0.060 *μ*M of oligo was titrated with ~0–50 *μ*M of protein, and a binding isotherm was generated. *K*_*d*_ values for each experiment are listed on the top left of each binding isotherm.

**Figure 5:**
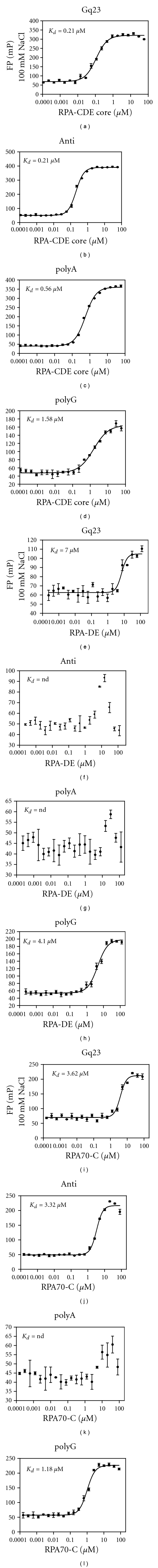
FP ssDNA binding assays for RPA-CDE-core, RPA-DE and RPA-C at 10 mM NaCl. Representative binding isotherms are shown. (a)–(d) RPA-CDE-core, (e)–(h) RPA-DE, and (i)–(l) RPA-C. The layout of the figure is the same as described in Figure 4. All experiments were performed at a lowered NaCl concentration to observe binding by the low-affinity RPA constructs and for comparison with RPA-CDE-core.

 DNA binding studies were performed to verify and understand the SELEX results. Several aspects of these experiments were carefully designed. In order to deconvolute the interactions of RPAs domains with Gq23, the TC-rich complement of Gq23 (Comp), polyA, and polyG were used as controls. It is noteworthy that the interactions of polyA and polyG with RPA are rarely studied because RPA prefers pyrimidine-rich sequences. Five different RPA constructs were used (i) full-length RPA, (ii) RPA-AB, (iii) RPA-CDE-core, (iv) RPA-DE, and (v) RPA-C. FP was selected as the method used to study the binding of RPA and its domains to various ssDNA sequences. The advantages of the FP binding assay are that (i) it is a direct and rapid assay that does not require radioactivity or gel electrophoresis and (ii) the reaction conditions can be easily varied to obtain equilibrium binding conditions. For example, electrophoretic mobility shift assays (EMSAs) performed with RPA, RPA-AB, and RPA-CDE-core indicated binding but it was hard to extrapolate and compare binding constants due to smearing (data not shown). One disadvantage that FP has over traditional EMSAs is that higher concentrations of ssDNA are needed to obtain an optimal fluorescence signal, and therefore the amount of protein needed is proportionally higher. For high affinity DNA binding proteins like full-length RPA, stoichiometric binding conditions occur in assays when the DNA concentration is equal to or higher than the dissociation constant [51]. Under stoichiometric conditions, the binding of DNA is not in equilibrium between the free and bound state but is pushed towards the bound state and the measured dissociation constant is underestimated. This makes it impossible to measure the real binding constant and masks the differences between the proteins and various ssDNA ligands. To obtain equilibrium binding and to overcome these problems, the assay was performed under conditions that lower the affinity of RPA for ssDNA, such as increasing the salt concentration. Binding of RPA and the various domains to the four ssDNA sequences was performed over a range of salt concentrations (10–1500 mM NaCl depending on the protein construct). Averages from all experiments are given in Table 1 with stoichiometric conditions underlined and specific examples of experiments are given in Figures 4 and 5. In the studies described below, for each protein construct the data is interpreted by comparing binding constants of the different ssDNA ligands, at the salt concentration where equilibrium binding is observed. 

Previous binding studies showed that for heterotrimeric RPA equilibrium binding occurs at a concentration of 1.25–1.5 M KCl [51]. In this study, in reactions containing 100 mM NaCl, binding of RPA to Gq23, Anti, and polyA sequences is stoichiometric for all ligands and the measured *K*_*d*_ is underestimated and equal to the ssDNA concentration (0.060 *μ*M, Table 1) while the binding to polyG is at equilibrium with a K_d_ of 260 nM (Figures 4(a)–4(d)). Equilibrium binding occurs at 1250 mM NaCl for Gq23, and 500 mM for polyA. The affinity of RPA for Anti is so high that binding is stoichiometric even at 1500 mM NaCl (Table 1). These data show that RPA prefers pyrimidine-rich sequences as expected and favors Gq23 over the polypurine sequences. Next, similar binding experiments were performed with the different DBDs of RPA to deconvolute their ssDNA sequence preferences. For these deletion mutants of RPA, equilibrium binding occurs at physiological salt levels and the binding studies at higher salt give further information on the relative affinity of RPA domains for various DNA sequences.

The binding of RPA-AB was studied. Equilibrium binding at 100 mM NaCl indicated no significant difference between Gq23 and Anti sequences (*K*_*d*_ = 0.4 and 0.7 *μ*M), and these binding constants were ~3-fold higher than values obtained for polyA and polyG (Table 1; Figures 4(e)–4(h)). Increasing salt to 1250 and 1500 mM abolishes binding to Gq23 as well as to polyA and polyG but weak binding is still detected for the Anti sequence (Table 1). These data indicate that RPA-AB binds to ssDNA with more than 10-fold lower affinity than full-length RPA and prefers pyrimidine-rich sequences. These results confirm previous studies where the affinity of RPA-AB for a dT_30_ sequence was two orders of magnitude lower than RPA [53].

When it became available, RPA-CDE-core was used for all DNA-binding experiments because it is stable, can be purified and concentrated with ease, and has a long shelf-life. Control experiments showed that RPA-CDE and RPA-CDE-core bind and unfold ssDNA with similar affinity (Supplemental Figure S6). Equilibrium binding was detected at 100 mM for all four sequences (Figures 4(i)–4(l)). The affinity of RPA-CDE-core for Gq23 and Anti was similar to RPA-AB and 5–10-fold lower for polyA and polyG. Previously, RPA-CDE-core was shown to have a 3–10-fold lower affinity than RPA-AB for a mixed 31-nt sequence [54]. To further unravel the binding properties of this RPA-CDE-core to ssDNA, FP experiments were performed using RPA-DE (Figure 1) which revealed interesting differences. 

RPA-DE has lower affinity [21] and differential affinities for various ssDNA sequences. FP experiments were performed at 100 mM NaCl but no binding was detected (Table 1). At 10 mM NaCl, dissociation constants of ~4–7 *μ*M were measured for polyG and Gq23 and binding was not detectable for Anti or polyA (Figures 5(e)–5(h)). This is a significantly different result when compared with RPA, RPA-AB, and RPA-CDE-core (Figure 4). To be able to directly compare RPA-DE with RPA-CDE-core, FP experiments with RPA-CDE-core at 10 mM NaCl were performed (Figures 5(a)–5(d)). Under these conditions, the binding of RPA-CDE-core to all four sequences was easily detected, whereas RPA-DE was only able to bind Gq23 and polyG. Overall, these results indicate that RPA-DE contributes significantly to the selection of the G-rich sequences obtained with SELEX. 

The individual contribution of RPA-C in ssDNA binding has not been studied because RPA1, when not in a complex with RPA2 and RPA3, is insoluble and cannot be purified. To study RPA-C, a new construct was designed by careful study of PDB entry 1L1O. It was engineered to destroy the heterotrimer interface, to be soluble, and to keep DBD-C with its zinc-finger intact. Binding was not detectable at 100 mM NaCl (Table 1). FP analysis (Figures 5(i)–5(l)) at 10 mM NaCl measured similar binding affinities (K_d_ ~ 3 *μ*M) for both Gq23 and Anti sequences with a slightly higher affinity for polyG (K_d_ ~ 1 *μ*M). This indicates that RPA70-C is truly a “universal binder” displaying very similar binding affinities for G-rich and TC-rich sequences, unlike RPA-DE which displays a preference for Gq23 and polyG, but not the TC-rich Anti sequence (Figures 5(e)–5(h)).

### 3.4. Deconvolution of which RPA Domains Unfold the G-Quadruplex

**Figure 6:**
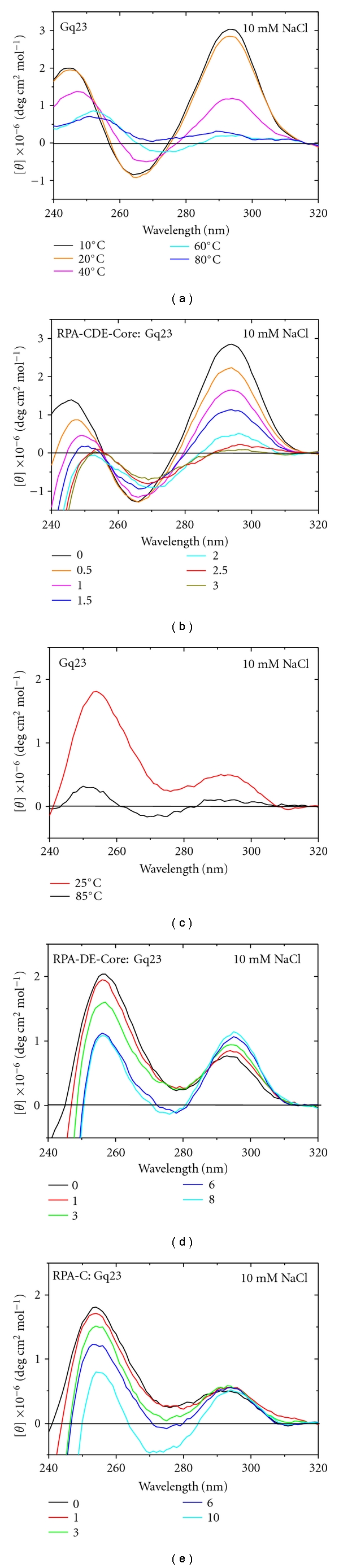
CD experiments for Gq23 oligonucleotide melting and protein titrations with RPA-CDE, RPA-DE, and RPA-C. (a) Melting of Gq23, (b) Titration of Gq23 with RPA-CDE at varying protein:ssDNA ratios. Peak at ~292 nm indicates the presence of an antiparallel G-quadruplex. Parts (a) and (b) were collected in Buffer A with 100 mM NaCl. (c) Melting of Gq23 in 10 mM NaCl, (d) Titration of Gq23 with RPA-DE, and (e) Titration of Gq23 with RPA-C. Parts (c–e) were collected in Buffer A with 10 mM NaCl. Note, spectra contain both antiparallel and parallel (254 nm) G-quadruplex peaks [10].

 Since the strong CD signals for proteins (Supplementary Figure S2) occur at a different wavelength range than those of ssDNA (190–240 nm versus 250–310 nm), it is possible to study the impact of protein binding on the structure and folding of ssDNA [55]. First the melting of Gq23 with temperature was studied. With increasing temperature, unfolding of Gq23 is indicated by the decrease in the peak at 292 nm and it is completely unfolded at ~60°C (Figure 6(a)). The unfolding of Gq23 by protein binding was studied next. When RPA-CDE-core was titrated against Gq23, the peak at 292 nm decreased and was complete at a molar ratio (RPA-CDE-core:Gq23) of 2 (Figure 6(b)). This indicates that RPA-CDE-core binds and unfolds the G-quadruplex as was previously observed for full length RPA [29]. Similar results were obtained for 100 mM NaCl (Figure 6(b)) and at 10 mM NaCl (data not shown). As a control, titration experiments were performed with TC23 titrated with RPA-CDE and no significant changes were seen in the conformation of the oligonucleotide (Supplemental Figure S5). This indicates that the TC23 remains a random coil when bound by RPA-CDE. Interestingly, the spectra of RPA-CDE-core bound to Gq23 show unfolding upon protein binding but do not indicate formation of a random coil. This implies that the structure of Gq23 when bound to RPA-CDE-core is different than a random coil, pyrimidine-rich structure. 

A similar experiment was conducted with RPA-DE and Gq23. Here, the reaction conditions were adjusted to ensure that RPA-DE can bind to Gq23 by lowering the salt concentration to 10 mM NaCl. Gq23 forms a G-quadruplex with both parallel and antiparallel peaks in this reaction condition (Figures 3(b) and 6(c)). CD spectra taken at 25 and 85°C show how the parallel and antiparallel peaks melt of Gq23 in 10 mM NaCl (Figure 6(c)). Interestingly, when the amount of RPA-DE was increased (Figure 6(c)), the G-quadruplex structure was not completely unfolded. The peak at 292 nm increased significantly with an increase in protein : DNA ratio, and the peak at 254 nm decreased but did not completely unfold. There is an isoelliptical (isosbestic) point at ~287 nm (Figure 6(d)) that indicates the two species are in equilibrium and the antiparallel form is favored with increasing protein. At a molar ratio of 8 : 1 (RPA-DE : Gq23), the peaks at 254 and 292 nm were of similar magnitude. These data indicate that the antiparallel conformation of the G-quadruplex was stabilized by RPA-DE in the absence of RPA-C. 

Since RPA-DE and RPA-C have similar binding affinities, but RPA-C binds Gq23 and Anti sequences equally, a similar CD experiment was performed with RPA-C. Here the reaction conditions were again adjusted to 10 mM NaCl to ensure binding. A similar decrease in the peak at 254 nm was observed with increasing protein : DNA molar ratio, but no change was seen in the peak at 292 nm (Figure 6(e)). Here, the addition of protein does not favor the antiparallel component. In conclusion, RPA-DE binding to Gq23 stabilizes the antiparallel peak at 292 nm but RPA-C does not.

## 4. Discussion

The diverse nature of RPA binding to ssDNA has been explored by several groups. However, so far the data are limited since most studies on RPA, and its domains, have been performed using primarily poly-pyrimidine ssDNA sequences. In this paper, the specific ssDNA sequences preferred by the DBDs of RPA were studied. An interesting SELEX result was obtained with RPA-CDE which selected a 20-mer G-rich sequence that formed an intramolecular G-quadruplex. The extensive binding studies in this work indicate that DBDs-A, -B, and -C of RPA contribute to the “universal binder” functions of RPA. With a soluble form of RPA-C, the binding characteristics of DBD-C alone were characterized. Binding affinity, with the RPA-C construct whose binding has not been studied previously, indicates that this construct binds to TC-rich and G-rich sequences alike with a binding constant ~3 *μ*M. DBD-D and -E appear to contribute to a more specialized function for binding G-rich sequences. 

CD studies showed that full length RPA and RPA-CDE core (data not shown and Figure 6(b)) bind and unfold the G-quadruplex. RPA-DE, on the other hand, stabilized the G-quadruplex secondary structure (Figure 6(c)), a result that is different from the binding of RPA-C alone to Gq23 (Figure 6(d)). Taken together, it is likely that RPA-DE can recognize the G-quadruplex fold and in the context of the RPA heterotrimer, the G-quadruplex becomes unfolded, after which point RPA-DE could bind to the unfolded G-rich ssDNA. 

**Figure 7:**
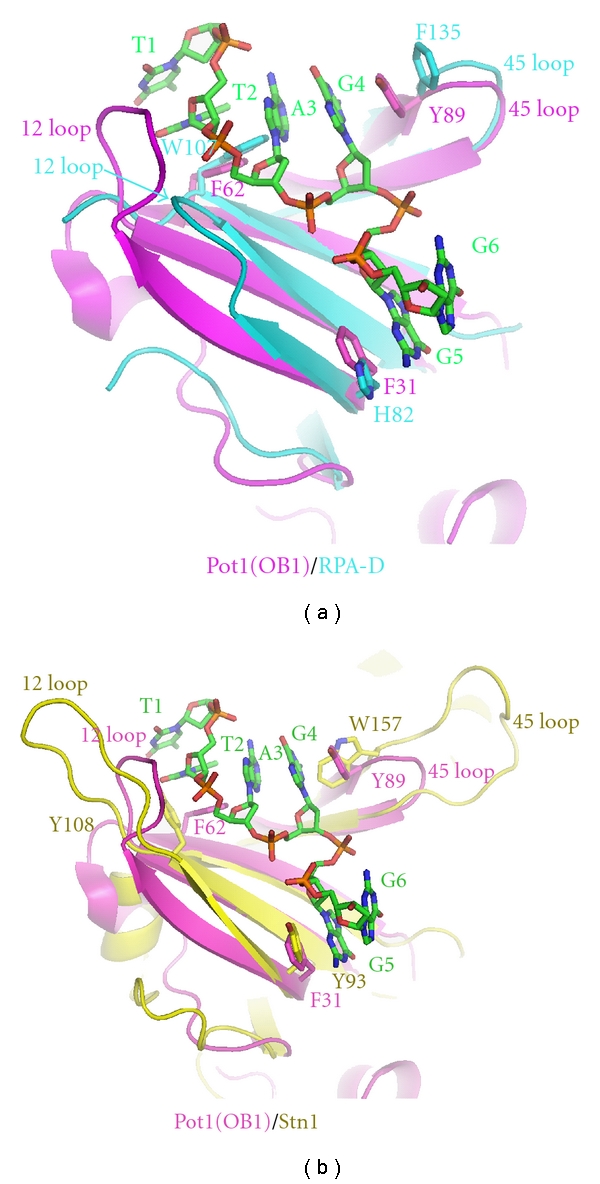
Comparison and prediction of ssDNA-binding sites. (a) Superposition of RPA-D (cyan) of with Pot1 (magenta). Alpha carbons from residue ranges of RPA2 residues, 130–142, 74–83, and 101–107 were superimposed with Pot1 (OB-1), 84–96, 23–32, and 56–62, respectively, with an RMSD of 0.8 Å. Pot1 residues 6–145 and RPA residues 43–171 are indicated as ribbons. DNA bases 1-6 (5′-TTAGGG-3′) from the Pot1 crystal structure are shown in green. (b) Superposition of Stn1 (yellow) with Pot1 (magenta). Alpha carbons from residue ranges 152–155, 122–127, 110–115, and 87–94 of Stn1 were aligned with residues 84–89, 56–61, 45–50, and 25–32 of Pot1, respectively, with an RMSD of 1.9 Å. Aromatic residues involved in stacking interactions from all proteins are shown as sticks. The superpositions of PDB entries 1XJV, 1QUQ, and 3KF8 were performed using ccp4i (LSQMK) [11] and displayed with Pymol (http://pymol.sourceforge.net/).

RPA and Pot1 are both ssDNA binding proteins with OB-folds which recognize, bind, and unfold G-quadruplex structures [26, 42, 46, 47]. There is no structural information available for how RPA-DE binds ssDNA. Therefore, to better understand the specialized function of RPA-DE, its structure was compared to the available crystal structure of Pot1 bound to telomeric ssDNA (5′-TTAGGGTTAG-3′) [7, 43]. In this analysis, RPA2 DBD-D superimposed well with OB-1 of Pot1 (Figure 7(a)). RPA-D has a shallow surface similar to Pot1 where the groove is wide enough to encompass larger purine bases. Pot1 aromatic residues F31, F62, and Y89, stack with the nucleotide bases G5, T2, and G4, respectively, and are important for binding [43] (Figure 7). Aromatic residues are also conserved at these positions in RPA-D and this predicts that H82, W107, and F135 are important for binding unfolded ssDNA. H82 is oriented similarly to F31 and stacks well with base G5 of the ssDNA. W107 aligns well with F62 to contact the DNA at base T2. F135 is in the same orientation as Y89 and the conformational change of loop L45 upon DNA binding would cause it to stack with base G4 (Figure 7(a)). From the superposition, it is clear that these residues are at the DNA binding interface and are in the correct position and orientation to stack with DNA bases although a conformational change of the protein (and the ssDNA) probably occurs.

Stn1 is an OB-fold protein that forms a complex with Cdc13 and Ten1, binds to telomeric repeats in yeast, and possesses sequence and structural homology to RPA2 [45]. To see if the aromatic triad was conserved in Stn1 as well, residues from the OB-fold of *C. tropicalis* Stn1 were superimposed with OB-1 of Pot1 (Figure 7(b)) [43, 45]. Aromatic residues Y93, Y108, and W157 of Stn1 were present in the same vicinity as residues F31, F62, and Y89 of Pot1 and are available for stacking interactions with bases G5, T2, and G4. This comparison predicts this surface as a DNA-binding site on Stn1. 

**Figure 8:**
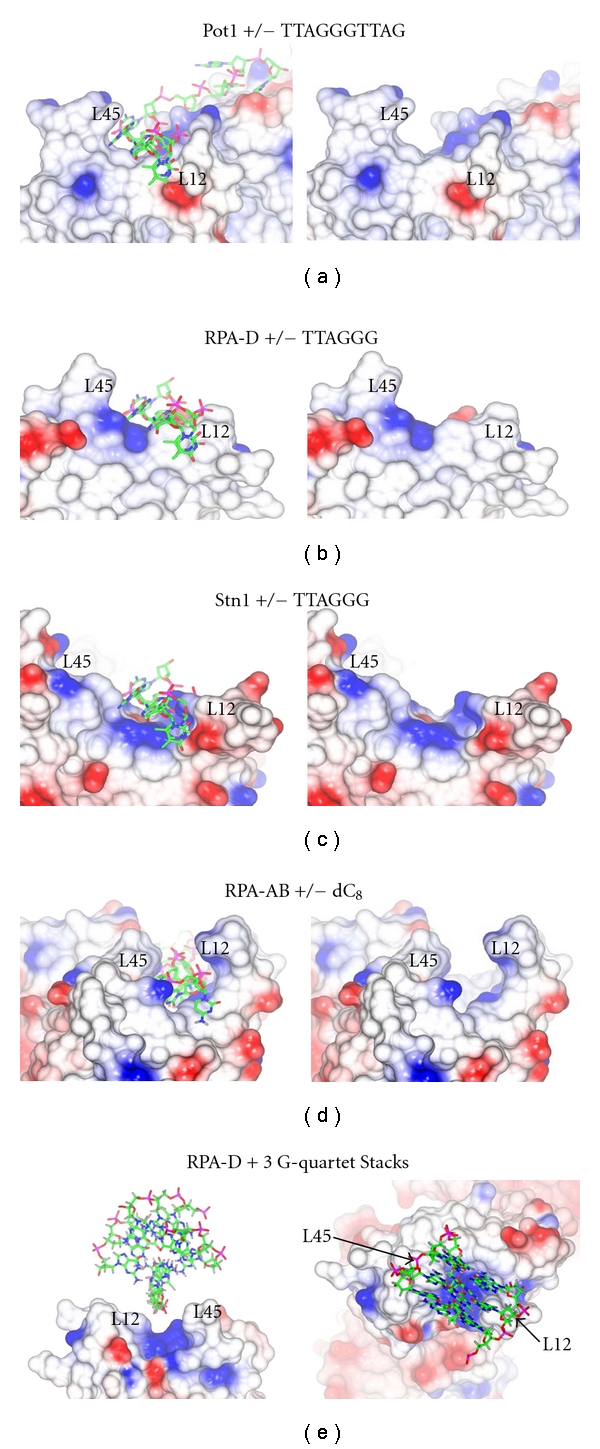
Electrostatic surface potential of the proteins (a) Pot1, (b) RPA-D, (c) Stn-1, and (d) RPA-AB are depicted with ssDNA (left) and without ssDNA (right). The ssDNA from the Pot1 crystal structure is shown here as a reference point to indicate the known/predicted binding site for the surfaces of Pot1, RPA-D, and Stn1. RPA-AB is shown with dC_8_ bound (PDB 1JMC). (e) Left, side view of the surface electrostatic potential of RPA-D rotated 180° about Y relative to Figure 8, with an antiparallel G-quadruplex (PDB ID: 2E4I, trimmed to include only the 3 stacks of G-quartets) displayed in the potential binding groove formed between L12 and L45; right, top view. All the figures were created using ccp4mg with −0.5 V (red) to +0.5 V (blue) [12]. For parts (b) and (e), the surface was calculated with PDB entry 1QUQ and included both RPA2 and RPA3 coordinates, but only RPA-D is displayed.

Next, the surface electrostatic potentials of Pot1, RPA-D, Stn1, and RPA-AB were compared with either ssDNA (5′-TTAGGGTTAG-3′) from the Pot1 model (Figures 8(a)–8(c)) or the ssDNA (dC8) from the RPA-AB model (Figure 8(d)) shown to mark the known/predicted ssDNA binding sites. For RPA-D, the coordinates for RPA-DE were used to calculate the electrostatic surface potential but only RPA-D is displayed (Figures 8(b) and 8(e)). All the proteins have an overall positively charged surface that electrostatically complements the negatively charged phosphate backbone of the ssDNA. From the Pot1 crystal structure, it is apparent that the ssDNA binding groove of Pot1 is wide enough and encompasses larger purine bases which bind and stack nicely in the groove formed by the loop between *β*-strands 1 and 2 (L12) and the loop between *β*-strands 4 and 5 (L45). The surface of RPA-D with the ssDNA from the Pot1 model (Figure 8(b)) indicates that loops L12 and L45 on RPA-D are short, making a binding pocket that is relatively shallow and wide as was seen with Pot1. Similarly, when Stn1 was displayed with ssDNA from the Pot1 model, the surface indicated a wide and shallow potential ssDNA-binding groove between loops L12 and L45 (Figure 8(c)), but L45 from Stn1 was much longer than L45 of Pot1 and RPA2-D and would probably change its conformation upon DNA binding. When the binding of RPA-AB to dC_8_ was compared to the potential G-quadruplex binding site of RPA-D, it was apparent that the grooves are very different where L12 and L45 form a deep, narrow ssDNA-binding pocket [56]. These differences are consistent with the differences in affinity and specificity between RPA-D and RPA-AB. 

Since the CD data indicate that RPA-D can stabilize the G-quadruplex structure without unfolding it and to further analyze the DNA binding groove on RPA-D, a mixed-parallel/antiparallel G-quadruplex molecule with 3-G-quartet stacks [10] was manually docked near the surface of RPA-D. From this, it was apparent that the binding groove of RPA-D can easily accommodate a folded G-quadruplex structure (Figure 8(e)). The potential binding groove formed between L12 and L45 with a highly basic surface potential, seen clearly in the top view, is the same width as the three G-quartet stacks (Figure 8(e), right). This is consistent with the CD data that (Figure 6(d)) indicates that RPA-DE does not unfold the G-quadruplex for binding, but stabilizes the G-quartet stacks formed. 

These similarities in structure and DNA-binding properties between RPA-D and the DBDs of Pot1 and Stn1 suggest that these domains have evolved to recognize related DNA structures. Hence, the more specialized function of RPA-D could be to recruit RPA to loci with G-quadruplex-forming sequences, including telomeres and promoters of the c-myc, HIF-1*α*, bcl-2, and c-kit genes. At these locations, RPA might perform other specialized functions unrelated to DNA replication, such as telomere protection and transcriptional regulation. Conversely, the “universal binder” functions of the other DBDs and their ability to melt G-quadruplexes would better support RPA's primary function in DNA replication as the primary eukaryotic ssDNA-binding protein. Several studies have now reported findings consistent with the notion of a separate function for RPA at telomeres. This speculation requires further study and scrutiny but allows for the combination of previous data and the data presented in this paper to further unravel the multiple roles for RPA in a cell.

## 5. Conclusions

In the experiments presented in this paper, SELEX experiments indicated that RPA-CDE bound preferentially to a G-rich, G-quadruplex forming sequence. Using a combination of FP and CD experiments, the domains of RPA were systematically evaluated for DNA-binding and their ability to unfold or stabilize the G-quadruplex DNA. In summary, RPA-AB binds TC-rich DNA, RPA-C is a universal binder (binding to pyrimidine and purine-rich sequences alike), and RPA-DE binds G-rich DNA. These data reveal a mechanism for how RPA can bind to a multitude of DNA sequences during its function in DNA replication as well as elucidates a potential mechanism for how RPA can bind to G-rich regions in the DNA capable of forming complex secondary structures.

## Supplementary Material

Supplementary Figure S1: SDS-PAGE stained with Coomassie Blue after protein purification.
Supplementary Figure S2: Circular dichroism spectra of proteins alone at varying temperatures.
Supplementary Figure S3: Proteins bind specifically to ssDNA.
Supplementary Figure S4: Abundance of trinucleotides from SELEX with RPACDE.
Supplementary Figure S5: TC23 and binding to protein constructs.
Supplementary Figure S6: Characterizaion of RPA-CDE binding to Gq23.

## Figures and Tables

**Table 1:** Summary of dissociation constants obtained from FP binding assays^a,b^.

	Gq23	Comp	polyA	polyG
	*K*_*d*_ (*μ*M)	*K*_*d*_ (*μ*M)	*K*_*d*_ (*μ*M)	*K*_*d*_ (*μ*M)
**RPA**				
NaCl (mM)				
100	**0.05**	**0.07**	**0.07**	0.26±0.06
500	0.04 ± 0.02	0.06 ± 0.01	0.17 ± 0.02	nd
1250	0.37 ± 0.08	**0.07 **	nd	nd
1500	0.85 ± 0.16	**0.07**	nd	nd

**RPA-AB**				
NaCl (mM)				
100	0.44 ± 0.06	0.66 ± 0.21	1.83 ± 0.39	1.54 ± 0.35
500	3.28 ± 0.68	1.79 ± 0.36	nd	nd
1250	nd	2.65 ± 0.15	nd	nd
1500	nd	2.45 ± 0.33	nd	nd

**RPA-CDE-core**				
NaCl (mM)				
10	0.16 ± 0.05	0.22 ± 0.01	0.53 ± 0.03	1.72 ± 0.13
100	0.55 ± 0.09	0.42 ± 0.04	5.88 ± 0.72	11.01 ± 0.97
500	11.92 ± 0.12	0.75 ± 0.02	nd	nd
1250	nd	2.81 ± 0.16	nd	nd

**RPA-DE**				
NaCl (mM)				
10	6.68 ± 0.23	nd	nd	4.30 ± 0.20
100	nd	nd	nd	19.18 ± 3.68

**RPA-C**	
NaCl (mM)				
10	3.62 ± 0.26	3.32 ± 0.02	nd	1.18 ± 0.18
100	nd	nd	nd	nd

^a^Values reported here are averages from two separate experiments where each data point was performed in triplicate. Error values obtained were between 5 and 10%.

^b^Data collected under stoichiometric conditions is underlined and the *K*_*d*_ is underestimated.

nd = not determinable (the experiments were performed but binding was not detectable). The *K*_*d*_s cannot be estimated because at higher protein concentrations no binding is detected, and binding saturation is not obtained.

## Abbreviations

CD: Circular dichroism
DBD: DNA binding domain
DTT: Dithiothreitol
EDTA: Ethylenediaminetetraacetic acid
EMSA: Electrophoretic mobility shift assay
FAM: Fluorescein
FP: Fluorescence polarization
IPTG: Isopropyl *β*-D-1-thiogalactopyranoside
Pot1: Protector of telomeres 1
RPA: Replication protein A
SELEX: Systematic evolution of ligands by exponential enrichment
ssDNA: Single-stranded DNA.
