# Supplementary material for: Identification of the DNA-Binding Domains of Human Replication Protein A That Recognize G-Quadruplex DNA
Source: J Nucleic Acids. 2011 May 21;2011:896947. doi: 10.4061/2011/896947 (PMC3136212; doi:10.4061/2011/896947)
Supplement: Supplementary file 1 — Supplementary Figure S1: SDS-PAGE stained with Coomassie Blue after protein purification. Supplementary Figure S2: Circular dichroism spectra of proteins alone at varying temperatures. Supplementary Figure S3: Proteins bind specifically to ssDNA. Supplementary Figure S4: Abundance of trinucleotides from SELEX with RPACDE. Supplementary Figure S5: TC23 and binding to protein constructs. Supplementary Figure S6: Characterizaion of RPA-CDE binding to Gq23. [file 896947.f1.pdf]

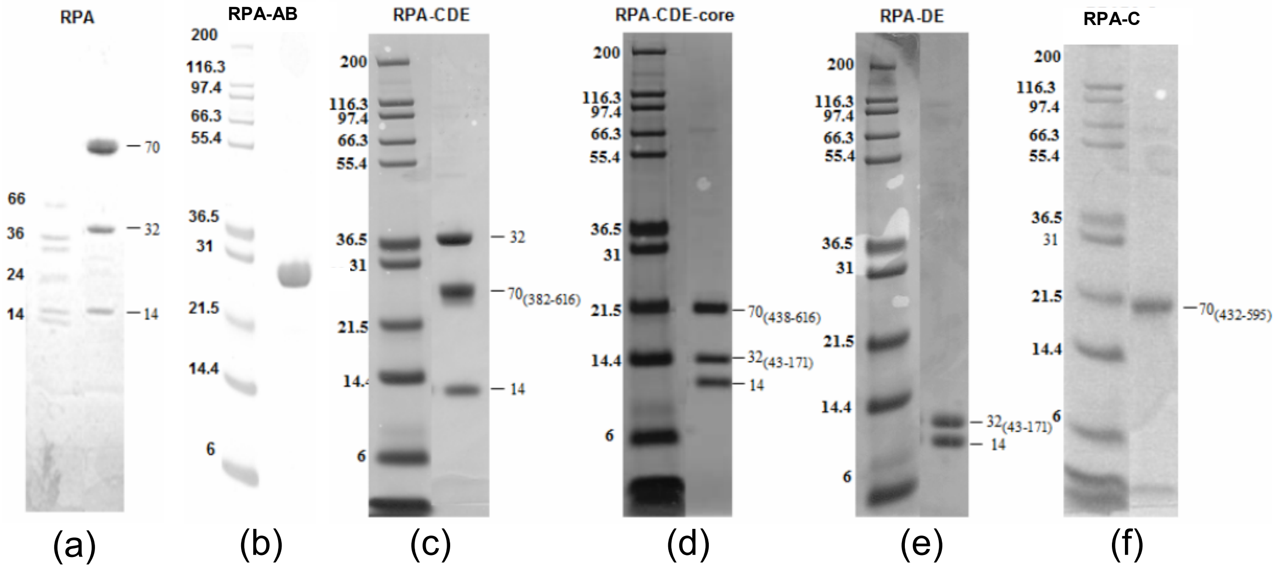

Supplemental Figure S1: SDS-PAGE stained with Coomassie Blue after protein purification. (a) RPA; (b) RPA-AB; (c) RPA-CDE; (d) RPA-CDE core; (e) RPA-DE; and (f) RPA-C purified to homogeneity from *E. coli* using column chromatography as described in the materials and methods section.

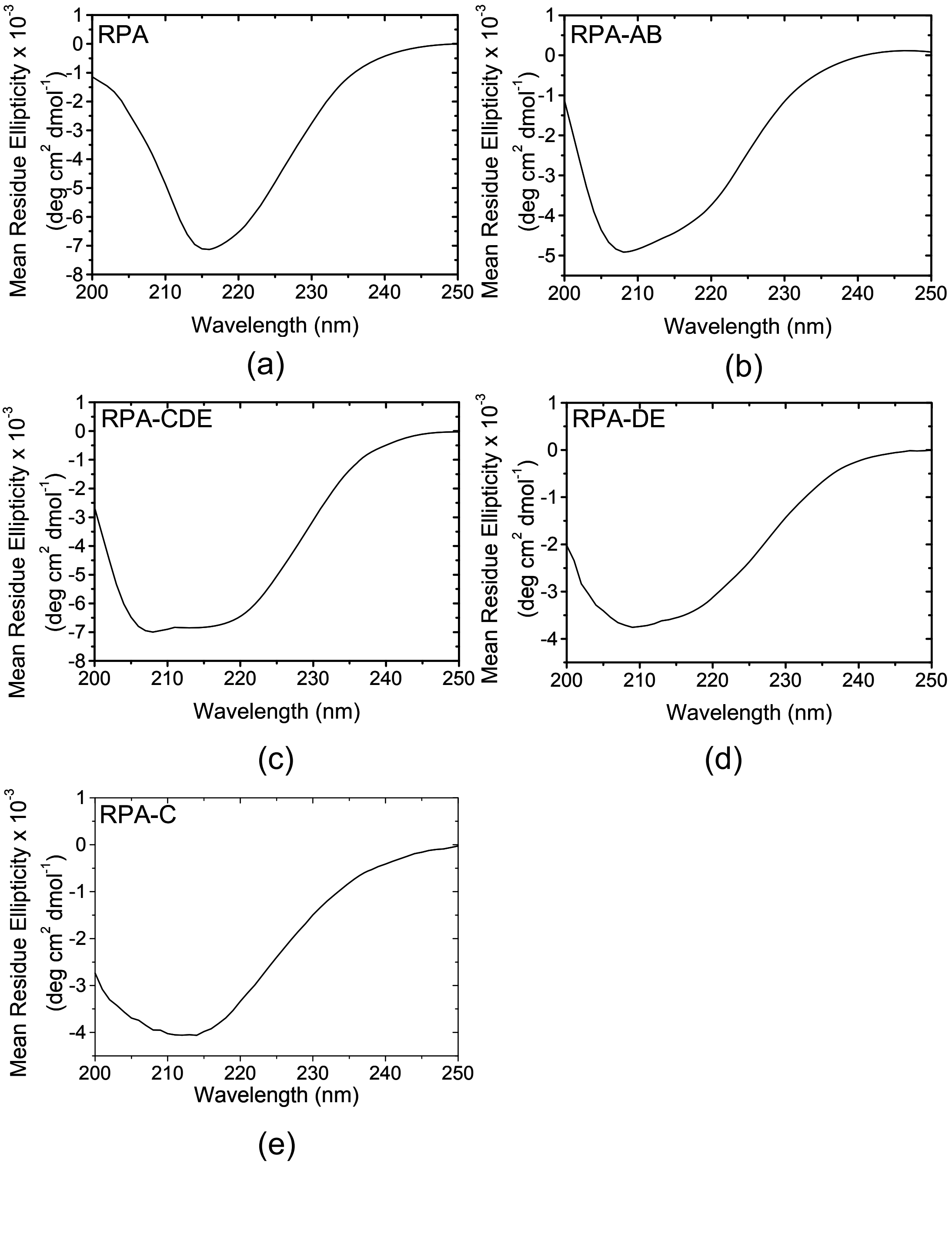

RPA
RPA-AB
RPA-CDE
RPA-DE
RPA-C
Mean Residue Ellipticity x 10^{-3}
(deg cm^2 $dmol^{-1}$)
Wavelength (nm)
(a)
(b)
(c)
(d)
(e)

Supplemental Figure S2. Circular dichroism spectra of proteins alone at varying temperatures: (a) Spectra of RPA alone. (b) Spectra of RPA-AB. (c) Spectra of RPA-CDE. (d) Spectra of RPA-DE. (e) Spectra of RPA-C. All spectra recorded are the average of 5 scans. In all the above graphs, the spectra at 25 ˚C are very similar to these at the physiological 37 ˚C temperature. This is important because all binding reactions were carried out at room temperature. CD spectra of α-helices are indicated by negative bands at 208 nm and 222 nm. Negative bands at 218 nm are indicative of β-sheet like structures. The deconvolution program CDSSTR using the Dichroweb server, that yielded best fit results was used in estimation of secondary structure [1]. CD spectra of the three proteins are in accordance with the secondary structures predicted by published 3D structures of these proteins or close relatives. The results for trimeric RPA indicates 6% α-helical content, 38% β-sheet, 23% turns and 33% disorder. CD spectra of full length RPA have been reported previously using similar analysis under slightly different buffer conditions [2]. Analysis of available crystal/ NMR structures of RPA also reveal that the majority of RPA is in the form of β-sheet (~30 %) [2]. Similarly for RPA-AB, this analysis indicates 7% α-helix, 37% β-sheet, 24% turns and 32% disorder. The crystal structure of RPA-AB residues 181-422 (slightly shorter than the construct used here which is residues 181 - 441) indicates 16% α-helix, and 40% β-sheet [3]. Again, a majority of the construct is in the form of β-sheets. RPA-CDE data indicate 16% α-helix, 20% β-sheet, 19% turns, and 44% disorder compared to the truncated construct from the crystal structure of the trimer core [4] that displays 21% α-helical content, and 36.5% β-sheet content. It should be noted here that RPA-CDE contains portions of RPA32 that are not solved in the trimer-core crystal structure. A similar analysis was performed for RPA-DE. CD data with RPA-DE indicate 10% helical content with 47% β-sheet regions. From the crystal structure of RPA-DE, the secondary structural analysis reveals 13% helical regions with 2 helices over 17 residues and 41% β-sheet with 9 strands formed by 54 residues. RPA70-C after purification contains a small amount (1 M) urea to stay in soluble in solution. This low urea concentration is not considered denaturing and the protein still remains folded. According to CD data obtained the protein contains 5% helical regions, 40% β-sheet, 22% turns and 32% disorder. There is no NMR or crystal structure available for this domain alone, since the construct has not been previously isolated or purified. Therefore, from these data it is evident that the recombinant proteins purified in solution here are properly folded and can be used in further in vitro assays.

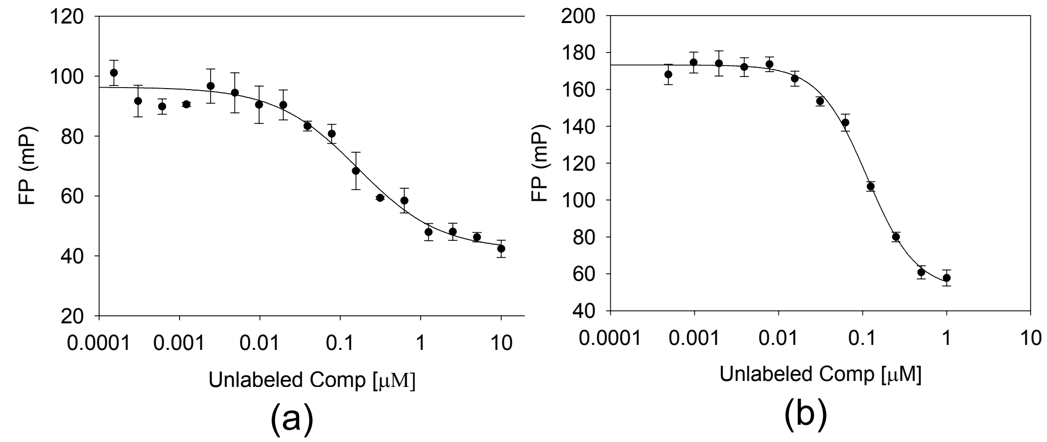

Supplementary Figure S3: Proteins bind specifically to ssDNA. (a) Competition FP experiments with RPA and (b) RPA-CDE core. In separate reactions, RPA and RPA-CDE core were combined with 60 nM 5' FAM labeled Comp oligo and allowed to incubate at RT for 15 minutes. This was followed by the addition of increasing concentrations (from 0 – 10 μM) of a competitor unlabeled Comp oligo. A decrease in polarization indicates that an unlabeled oligo with the same sequence can effectively compete off the labeled oligo, indicative of no non-specific binding (i.e. binding to the label).

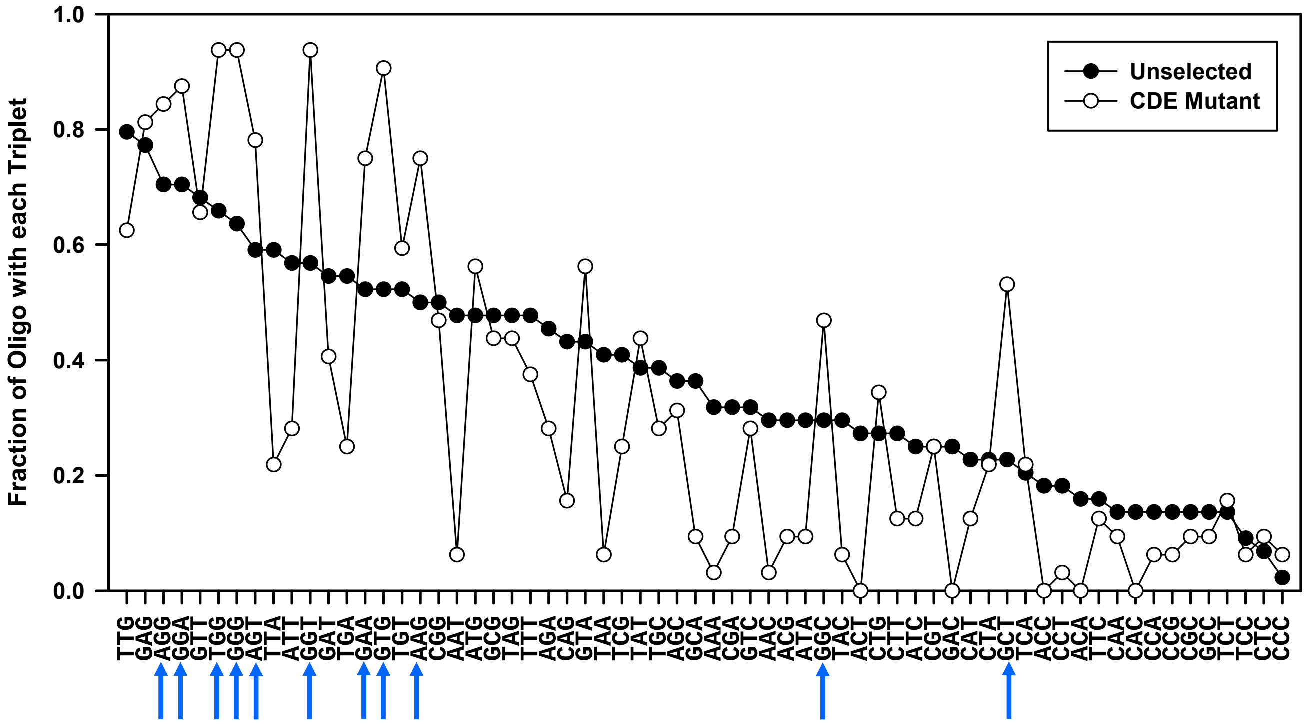

Supplemental Figure S4. Abundance of trinucleotides from SELEX with RPA-CDE. After 6 rounds of SELEX with RPA-CDE, the cloned sequences were analyzed by counting the frequency of molecules that contain each trinucleotide plotted against the 64 possible trinucleotides (on the x-axis). The x-axis is arranged so that the trinucleotides presented in the unselected, control pool (filled circles) are listed in order of decreasing frequencies. This baseline shows a slight bias towards dGTP and dTTP in the original pool. Sequence analysis of 32 sequences after selection with RPA-CDE (open circles) reveals higher frequency of trinucleotides that are G-rich (blue arrows). The trinucleotides with the highest frequency above background were used as an initial pattern to search for a larger consensus motif (see Fig. 2 of main paper).

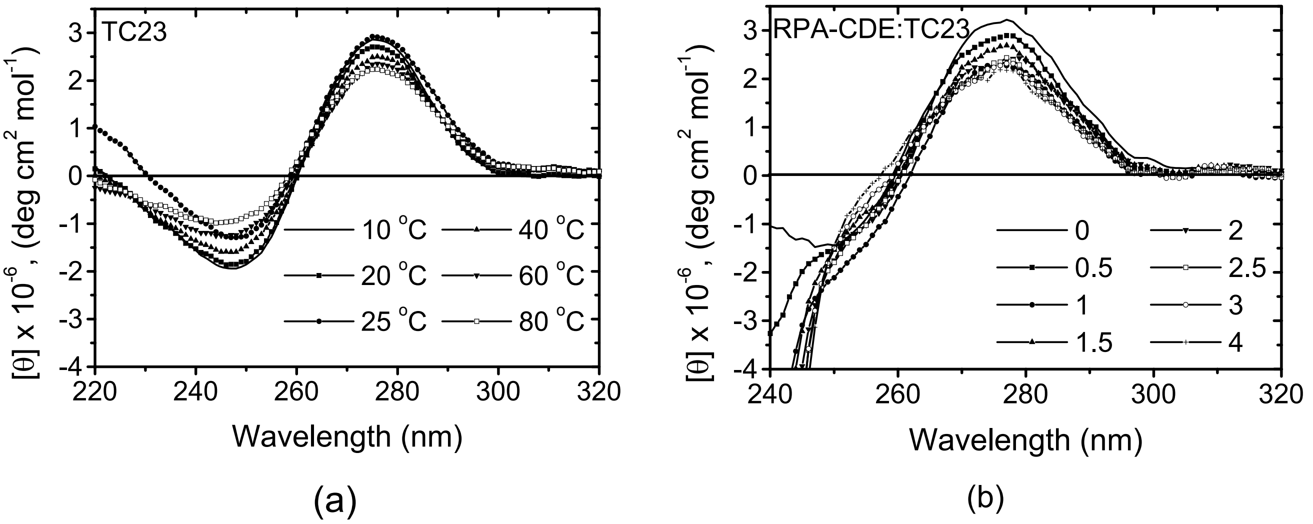

Supplemental Figure S5. TC23 and binding to protein constructs: (a) Spectra of TC23 at varying temperatures, 10 ˚C - 80 ˚C. TC23 forms a canonical random coil, and even at high temperatures of 80 ˚C. Random coil conformation is indicated by a peak at 280 nm and crossover at 260 nm. (b) Spectra of RPA-CDE titrated with TC23 at varying protein:ssDNA ratios. No change is seen in the conformation of the random coil but there is a slight perturbation of the crossover at 260 nm probably occurring due to changes in stacking interactions upon protein binding. Data were collected only to 240 nm because wavelengths <240 nm are dominated by protein secondary structure.

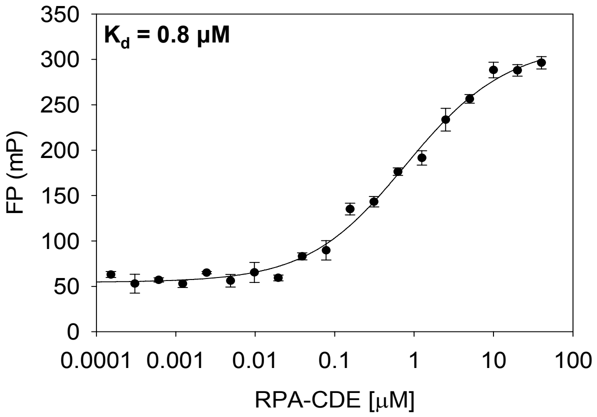

(a)

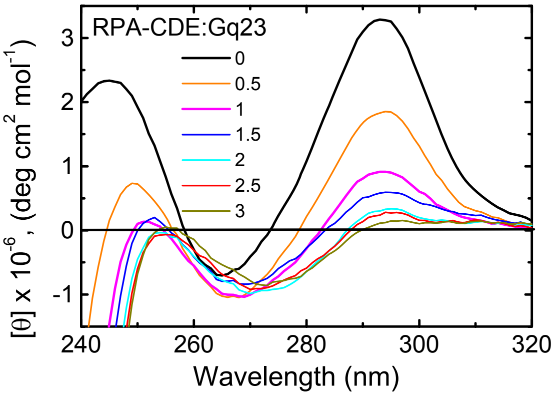

(b)

Supplementary Figure S6. Characterizaion of RPA-CDE binding to Gq23. (a) FP binding assay with RPA-CDE and Gq23 at 100 mM NaCl indicates a K_d similar to that of RPA-CDE core (~0.6 μM). K_d values for RPA-CDE and Comp sequence also at 100 mM NaCl was 0.2 μM also similar to that obtained with RPA-CDE core (~0.4 μM) (data for only Gq23 is shown above). (b) CD experiments on Gq23 with titration of RPA-CDE. The unfolding of the G-quadruplex by RPA-CDE is very similar to that by RPA-CDE-core (Fig. 6B). Parts A and B were collected in Buffer A with 100 mM NaCl.

References

1. N. Sreerama and R. W. Woody, "Estimation of protein secondary structure from circular dichroism spectra: Comparison of contin, selcon, and cdsstr methods with an expanded reference set," *Anal Biochem*, vol. 287, no. 2, pp. 252-260, 2000.
2. J. H. Fan, E. Bochkareva, A. Bochkarev, and D. M. Gray, "Circular dichroism spectra and electrophoretic mobility shift assays show that human replication protein a binds and melts intramolecular g-quadruplex structures," *Biochemistry*, vol. 48, no. 5, pp. 1099-1111, 2009.
3. A. Bochkarev, R. A. Pfuetzner, A. M. Edwards, and L. Frappier, "Structure of the single-stranded-DNA-binding domain of replication protein a bound to DNA," *Nature*, vol. 385, no. 6612, pp. 176-181, 1997.
4. E. Bochkareva, S. Korolev, S. P. Lees-Miller, and A. Bochkarev, "Structure of the RPA trimerization core and its role in the multistep DNA-binding mechanism of rpa," *Embo J*, vol. 21, no. 7, pp. 1855-1863, 2002.
